# Local and systemic oxidant/antioxidant status before and during lung cancer radiotherapy

**DOI:** 10.1080/10715760902942824

**Published:** 2009-06-11

**Authors:** Marika Crohns, Seppo Saarelainen, Hannu Kankaanranta, Eeva Moilanen, Hannu Alho, Pirkko Kellokumpu-Lehtinen

**Affiliations:** 1 Department of Oncology, University of Tampere, PO Box 607, FI-33101 Tampere, Finland; 2 Science Center, Pirkanmaa Hospital District, PO Box 2000, FI-33521 Tampere, Finland; 3 Department of Respiratory Medicine, Tampere University Hospital, PO Box 2000, FI-33521 Tampere, Finland; 4 Department of Pulmonary Medicine, University of Tampere, PO Box 607, FI-33101 Tampere, Finland; 5 Department of Respiratory Medicine, Seinäjoki Central Hospital, Hanneksenrinne 7, FI-60220 Seinäjoki, Finland; 6 Medical School, University of Tampere, PO Box 607, FI-33101 Tampere, Finland; 7 Department of Mental Health and Substance Abuse Services, National Institute for Health and Welfare, PO Box 30, FI-00271 Helsinki, Finland; 8 Research Unit of Substance Abuse Medicine, University and University Hospital of Helsinki, Helsinki, Finland; 9 Department of Oncology, Tampere University Hospital, PO Box 2000, FI-33521 Tampere, Finland

**Keywords:** Oxidative stress, BAL fluid, lung cancer, radiotherapy, antioxidants, nitrosative stress

## Abstract

To examine local and systemic oxidative status of lung cancer (LC) and oxidant effects of radiotherapy (RT), this study evaluated antioxidants and markers of oxidative and nitrosative stress in bronchoalveolar lavage (BAL) fluid and in the blood of 36 LC patients and 36 non-cancer controls at baseline and during and after RT for LC. LC patients had higher baseline serum urate, plasma nitrite and lower serum oxidized proteins than controls (*p* = 0.016, *p* < 0.001 and *p* = 0.027, respectively), but BAL fluid oxidative stress markers were similar. RT tended to raise some antioxidants, however, significant increases were seen in serum urate, conjugated dienes and TBARS (*p* = 0.044, *p* = 0.034 and *p* = 0.004, respectively) 3 months after RT. High urate at baseline may compensate against the oxidative stress caused by LC. RT shifts the oxidant/antioxidant balance towards lipid peroxidation, although the antioxidant defense mechanisms of the body appear to counteract the increased oxidative stress rather effectively.

## Introduction

Lung cancer (LC) is the leading cause of cancer-related deaths in the Western world [1]. LC is divided into two main groups: non-small cell lung cancer (NSCLC) and small cell lung cancer (SCLC). Treatment is based on the staging of the cancer and on the performance status of the patient. Surgery is the main treatment for limited disease. Advanced NSCLC is mainly treated with radiotherapy (RT) and chemoradiotherapy [2–4].

Radiation generates primary radicals by transferring energy to certain cellular components, e.g. water [5]. Reactive oxygen species (ROS) are formed in this process and they mediate the anti-tumour effects of RT [6]. A delicate situation occurs at the tissue level when oxidative stress is a desired effect against malignant cells; yet the amount of oxidative stress should be kept in balance to prevent permanent damage to normal cells.

An increasing number of studies have been published where bronchoalveolar lavage (BAL) has been used as a window to assess the oxidative status of the lungs, e.g. in asthma, pulmonary fibrosis, chronic obstructive pulmonary disease (COPD) and after exposure to ozone and diesel [7–12]. However, there are only a limited number of clinical studies involving BAL in LC patients. Melloni et al. [13] reported increased glutathione and reduced superoxide dis-mutase levels in BAL fluid of LC patients compared to non-cancer controls. The levels of vascular endothelial growth factor in BAL fluid are elevated in LC patients before and during radio-chemotherapy [14,15]. RT raises the concentration of interleukin-6 and TGF-β_1_ in the BAL fluid of LC patients [16].

The respiratory tract lining fluid (RTLF) contains a variety of antioxidant enzymes (superoxide dismu-tase, catalase, glutathione peroxidase and glutathione reductase), small non-enzymatic antioxidants (vitamin C, E, A and glutathione) and other compounds like albumin, ceruloplasmin and transferrin. RTLF plays an important role in protecting the cells from external oxidants, e.g. tobacco smoke and air pollutants. The fluid obtained in connection with therapeutic or diagnostic BAL provides a sample of the cellular and non-cellular components from the site of the respiratory epithelium itself [7, 11, 17].

To explore the RTLF status and the systemic oxidative stress, we determined the levels of a number of antioxidants (ascorbic acid, vitamin E, alpha-and gamma-tocopherol, urate, thiols, glutathione), the total antioxidant capacity (TRAP) and several parameters of oxidative and nitrosative stress (proteins, oxidized proteins, TBARS, conjugated dienes, nitrite, nitrite + nitrate) in BAL fluid and blood of lung cancer patients and non-cancer controls. These markers were evaluated at baseline and during and after RT of lung cancer. We also examined whether these markers predict adverse events, response to treatment and overall survival of LC patients.

## Material and methods

### Study groups and procedures

Thirty-six histologically or cytologically confirmed LC patients and 36 non-cancer controls were enrolled at the Department of Respiratory Medicine. The inclusion criteria for both groups were: Kar-nofsky performance status of ≥ 70% and no serious acute infection. The exclusion criteria were: serious cardiac, metabolic or hepatic disease, forced expiratory volume in 1 s (FEV_1_) ≤ 1.5 l, regular allopurinol or acetylcystein medication or gout. The controls were recruited among the patients referred for bronchoscopy due to prolonged cough. The characteristics of the two groups are shown in Table I.

**Table I:** Characteristics of lung cancer patients and controls. Values are means (SD; range) or numbers (percentages).

	Lung cancer patients (*n* = 36)	Controls (*n* = 36)
Age (years)	66.9 (9.1; 47–82)	50.8 (9.9; 18–75)
Males	29 (80.6)	16 (44.4)
BMI (kg/m^2^)	23.7 (3.2; 17.0–31.8)	26.3 (5.2; 18.5–39.2)
FEV1 (% of predicted)^a^	74.1 (11.8; 58.0–94.0)	89.4 (11.5; 65–108)
Smoking		
Non-smokers	2 (5.6)	18 (50.0)
Ex-smokers	10 (27.8)	6 (16.7)
Smokers	24 (66.7)	12 (33.3)
Pack-years of smokers or ex-smokers	35.3 (23.0; 5.0–96)	28.5 (17.7; 7.5–68)
Histology		
Squamous cell cancer	33 (91.7)	
Adenocarcinoma	1 (2.8)	
Small cell cancer	2 (5.6)	
Stage of lung cancer		
IB	1 (2.8)	
IIIA	6 (16.7)	
IIIB	12 (33.3)	
IV	17 (47.2)	
Karnofsky performance status		
70	4 (11.1)	0 (0)
80	8 (22.2)	0 (0)
90	15 (41.7)	3 (8.3)
100	9 (25.0)	33 (91.7)

a *n* = 13 and *n* = 20.

Neither the patients nor the controls had taken vitamin or herbal supplementation within 3 months prior to the study. Data on smoking, other diseases, medication and symptoms were collected on a standardized questionnaire modified from the ATBC (Alpha-Tocopherol, Beta Carotene Cancer Prevention Study) study [18]. Pre-treatment evaluation of the LC patients consisted of a physical examination, chest radiography, bronchoscopy, chest and upper abdominal computerized tomography, urinanalysis, full blood count and serum chemistry. Abdominal sonography and bone scintigraphy were performed as needed. Smokers were defined either as current smokers or as smokers who had stopped smoking less than 6 months previously, ex-smokers as subjects who had stopped smoking more than 6 months ago and non-smokers had never smoked. The lifetime cigarette consumption was expressed as pack years (cigarette packs smoked/day × years smoked).

This study was conducted according to the guidelines of the Declaration of Helsinki. Written informed consent was obtained from each participant to a study protocol approved by the ethics committee of the Tampere University Hospital.

### Bronchoscopy and BAL samples

All patients and controls underwent bronchoscopy and BAL as a diagnostic procedure. Patients receiving RT underwent a second bronchoscopy and BAL 2 weeks after start of RT (at 18–22 Gray). During RT BAL was performed on the irradiated lung and on the same segment as at diagnosis, if possible. All bronchoscopies were carried out by the same experienced bronchoscopist (SS) according to standardized methods [19,20]. During bronchoscopy the subjects were awake and breathed spontaneously. All subjects received pre-medication with intramuscular atropine sulphate (0.7 mg) and topical lidocain anaesthesia to the nasal airways and posterior pharyngeal wall. A flexible bronchoscope was wedged into the segmental or subsegmental level of the left upper lobe or right middle lobe. Five-times 20 ml of sterile saline (37°C) mixed with Addiphos buffer (4% v/v, Fresenius Kabi, Uppsala, Sweden) was instilled through the bronchoscope. The fluid was immediately recovered by gentle suction after each instillation [19,20]. Due to ethical reasons, BAL was performed only on one side of the lungs at each bronchoscopy.

The BAL fluid samples were immediately protected from light and put on ice. The samples were centrifuged at 500 rpm for 15 min at 4°C and stored at −70°C until analysis. The samples for ascorbic acid analysis were mixed (1:10) with 5% meta-phoshoric acid and isoascorbate. Cells were stained with the May-Grűnwald Giemsa (MGG) and Papanicolau stains and fixed with 50% ethanol.

### Blood samples

All patients underwent laboratory testing at baseline. In addition to oxidative stress markers the tests included: full blood count, alkaline phosphatase, alanine transferase, aspartate transferase, creatinine, serum C-reactive protein, sodium and potassium.

The blood samples for oxidative stress markers were taken as follows: Peripheral venous blood samples were collected using a Venoject blood collection system (Terumo, Leuven, Belgium). Two tubes (10 ml each) of blood were obtained, one for serum preparation and one for plasma analysis. The serum samples were collected into sterile tubes and the samples intended for plasma analysis were collected to cooled, sterile tubes containing ethylenedia-minetetraacetic acid (EDTA). The samples were protected from light and centrifuged at 2800 g for 10 min after which the plasma specimen for ascorbic acid analysis was mixed (1:10) with 5% metaphoshoric acid and isoascorbate. The samples were frozen immediately and stored at −70°C until analysis.

Blood samples were collected of all patients at baseline. The second set of blood samples coincided with second bronchoscopy during RT at 18–22 Gray and the third 3 months after start of RT.

### Radiotherapy

Twenty out of 36 patients received RT as a treatment. Sixteen patients received RT based on three-dimensional CT-based treatment planning by Cad Plan (version 6.23, Varian Medical Finland, Varian Medical Systems Inc). Fractions (fr), usually of 2 Gray (Gy), were given five times a week. Four patients received palliative RT with two anterior-posterior fields 3 Gy/fr five times a week. The planning target volume (PTV) contained the primary tumour and adjacent lymph nodes with adequate margins. RT with 18 MV photons was applied by a linear accelerator (Varian Clinac 2100 C/D, Varian Medical Systems Inc., Palo Alto, CA) at the Radiotherapy Unit of the Tampere University Hospital. The mean radiation dose delivered was 46.9 Gray (range 30.0–60.0 Gy).

### Analyses

#### BAL fluid and plasma thiols

Thiols in 400 μl of BAL fluid or 100 μl of plasma were determined with Ellman's reagent as described [21]. The coefficient of variation between the series was 6.6%.

#### BAL fluid and plasma ascorbic acid

BAL and plasma ascorbic acid were determined in metaphosphoric acid (5%) stabilized samples by HPLC with electrochemical detection [22]. The coefficient of variation between the series was 5.5%.

#### BAL fluid and serum urate

BAL and serum urate were analysed by an enzymatic method (Thermo Fisher Scientific Oy, Vantaa, Finland) using uricase, perox-idase and ascorbate oxidase. The coefficient of variation between the series varied from 1.4–2.3% and the accuracy (bias) was + 1.4%, as assessed by an external quality programme (Labquality Ltd, Helsinki, Finland).

#### BAL fluid and plasma nitrite and Nox

Concentrations of nitrite and Nox (nitrite + nitrate) were measured by the ozone-chemiluminescence method [23,24]. The detection limit for nitrite was 0.2 μmol/L and 1.5 μmol/L for Nox.

#### Plasma tocopherols

Alpha- and gamma-tocopherols were analysed as described [25,26]. The coefficient of variation between the series was 5.2%. The results are expressed as mg/L.

#### Serum vitamin E

Serum vitamin E was measured by chromatographic methods as described [27].

#### Plasma glutathione

Total glutathione in the plasma (GSSG + GSH) was determined by an enzymatic recycling reaction [28].

#### Serum protein oxidation, diene conjugation, TBARS, TRAP

Protein carbonyl determinations were carried out as described [29]. Two different methods (diene conjugation, thiobarbituric acid reactive substances, TBARS) were used to estimate serum levels of lipid peroxides. Conjugated dienes were analysed spectro-photometrically at 234 nm [30]. For the analyses of TBA-reactive substances the absorbances were measured at 535 nm [31]. The antioxidant potential of the samples (total peroxyl radical trapping antioxidant potential, TRAP) was estimated by their potency to resist 2,2′-azobis(2-amidinopropane) (ABAP) induced peroxidation [32].

The concentrations of BAL and plasma thiols, BAL and plasma ascorbic acid, BAL and serum urate, BAL and plasma nitrite and Nox, serum vitamin E, plasma glutathione, serum protein oxidation, serum diene conjugation, serum TBARS and serum TRAP are expressed as mmol/L.

#### Serum proteins

Serum proteins were analysed by a colorimetric end-point measurement following the Biuret method [33,34]. The intra-assay coefficient of variation was 0.88% and the inter-assay 1.80%. The results are expressed as g/L.

#### BAL fluid cell counts

The total cell and differential cell counts were determined by microscopy of fixed BAL fluid samples. BAL fluid albumin was measured by nephelometry and proteins by colorimetry.

### Evaluation of adverse events and response to treatment

During and after the RT, all adverse events were evaluated according to the criteria of the World Health Organization (WHO) and Lent Soma Table [35,36]. The responses to treatment were evaluated according to the criteria of WHO [35].

### Statistics

The systemic and local oxidative stress markers were the primary variables of the study. The results are given as means or geometric means with 95% confidence intervals. The distributions of some oxidative stress markers were skewed to the right and were thus logarithmically (ln) transformed before analysis. The *t*-test for independent samples was used to compare patients and controls. Since there were differences in demographic variables, the adjusted comparison (ANCOVA) includes age and FEV_1_ (%) as continuous covariates and gender and smoking (smoker vs non-smoker) as random factors. The interaction terms between the explaining factors are not included in the multivariable models. The group comparisons are presented as mean difference or as a ratio (patients/controls) with 95% confidence intervals. The within-patient changes in oxidative stress markers were analysed with the *t*-test for paired samples and ANOVA for repeated measures. Spearman's and Pearson's correlations were calculated to express the associations between oxidative stress markers, age and pack-years of smoking. The *t*-test for independent samples was used to compare different demographic groups (grouping by smoking, gender, other diseases, stage of disease). The oxidative stress markers at baseline were divided into two groups (< median and > median) and the Kaplan-Meier method was applied to plot the survival curves. The log-rank test was used to compare the survival distributions. The survival times are given as medians with 95% confidence interval. The Mann-Whitney's U-test was used for variables with a non-normal distribution and the χ^2^-test was used for categorical variables. *p*-values of less than 0.05 were considered statistically significant. The statistical analyses were performed with the SPSS (release 15.0) software (SPSS inc. Chicago, IL).

## Results

### Patients and controls

Thirty-six LC patients entered to the study and 36 non-cancer patients served as controls (Table I). The diagnosis of LC was confirmed histologically in 27 of the patients and for the remaining nine patients the diagnosis was based on class V BAL cytology and imaging results. Twenty of the LC patients received radiotherapy, 15 of whom underwent a second bronchoscopy during RT. Two patients were treated only by surgery after the diagnosis, six received various chemotherapy regimens and nine received symptomatic treatment. Of these 16 patients only baseline samples were obtained.

The final diagnosis for the controls showed that none of them had cancer, three (8%) had asthma, two (6%) chronic bronchitis and mild chronic obstructive pulmonary disease (COPD), one (3%) COPD and one (3%) control patient was operated on for a benign papilloma. All other control patients (80%) were diagnosed as having idiopathic prolonged cough and had no further treatment.

The patient and control groups differed with respect to some major main characteristics (Table I) and thus the results were adjusted for age, FEV_1_, gender and smoking. There were no significant associations between any of the other diseases (cardiac disease, diabetes, chronic arrhythmia or rheumatic disease) with regard to oxidative stress marker levels.

The mean instilled BAL fluid volume was 111 ml for LC patients and 101 ml for controls (*p* = 0.033); the recovery volumes were 55.3 ml and 67.3 ml (*p* = 0.005), respectively, at first bronchoscopy. There were no statistically significant differences in the total cell counts of BAL fluid samples between the groups, but LC patients had a higher neutrophil count than controls (3% vs 1%; *p* = 0.002). There were no significant complications during or after the bronchoscopies.

### Systemic oxidative stress markers at baseline

The LC patients had significantly higher levels of urate (241 μmol/L, 95% CI 210–272 μmol/L vs 116 μmol/L, 95% CI 91–141 μmol/L, *p*<0.001) and nitrite (0.591 μmol/L, 95% CI 0.548–0.634 μmol/L vs 0.181 μmol/L, 95% CI 0.136–0.225 μmol/L, *p*<0.001) than the controls at baseline. The LC patients had significantly lower levels of thiols (303 μmol/L, 95% CI 283–322 μmol/L vs 364 μmol/L, 95% CI 338–390 μmol/L, *p*<0.001) and oxidized proteins (2.85 μmol/L, 95% CI 2.46–3.23 μmol/L vs 4.30 μmol/L, 95% CI 4.06–4.55 μmol/L, *p* < 0.001) than the controls at baseline.

**Figure 1:**
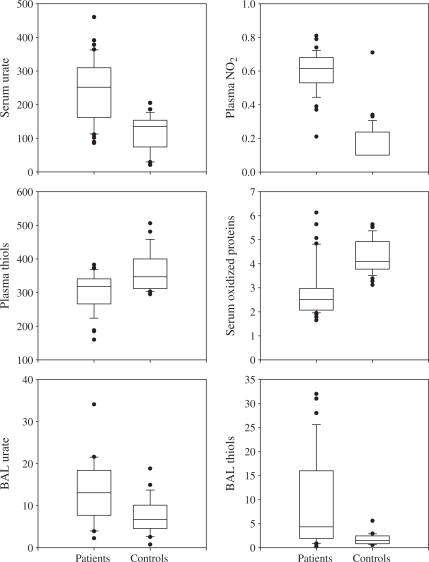
Box-plot figures of systemic and local oxidative stress markers (μmol/L) at baseline in patients and controls. The boxes indicate the lower and upper quartiles and the central line us the median. Whiskers above and below the box indicate the 90^th^ and 10^th^ percentile. Outliers are given as filled circles (•).

After adjustment for age, FEV_1_, gender and smoking, significant differences persisted for urate, nitrite and oxidized proteins (*p* = 0.016, *p* < 0.001 and *p* = 0.027, respectively), whereas significance was lost for thiol levels (*p* = 0.651) (Figure 1).

There were no significant differences in ascorbate (73.3 vs 78.3 μmol/L, *p* = 0.529), glutathione (geometric mean 0.79 vs 0.89 μmol/L, *p* = 0.636), vitamin E (26.7 vs 29.8 μmol/L, *p* = 0.264), conjugated dienes (51.8 vs 54.8 μmol/L, *p* = 0.420), TRAP (947 vs 963 μmol/L, *p* = 0.751), TBARS (4.64 vs 4.60 μmol/L, *p* = 0.855), Nox (28.6 vs 26.5 μmol/L, *p* = 0.482) or proteins (69.0 vs 72.0 g/L, *p* = 0.054) between the two groups at baseline (Table II).

**Table II:** Antioxidant variables at baseline as mean (95% CI) or geometric mean (95% CI) for patients and controls. The crude and adjusted group comparison is given as mean difference (95% CI) or as ratio patients/controls (95% CI). Measuring units: mg/L for plasma tocopherols, g/L for serum protein, μmol/L for other variables.

			Patients vs Controls			
			
			Crude^a^		Adjusted^b^	
				
	Patients	Controls	M (95% CI)	*p*	M (95% CI)	*p*
Plasma						
Alphatocopherol	7.39 (6.01–8.76)	NA				
Gammatocopherol	0.80 (0.55–1.04)	NA				
Ascorbate	73.3 (61.9–84.8)	78.3 (67.0–89.7)	−5.0 (−20.8 to 10.8)	0.529	6.8 (−37.1 to 50.7)	0.753
Thiols	303 (283–322)	364 (338–390)	−61 (−92 to −31)	<0.001	20.6 (−73.3 to 114.6)	0.651
Nitrite	0.591 (0.548–0.634)	0.181 (0.136–0.225)	0.41 (0.35 to 0.47	<0.001	0.32 (0.18 to 0.46)	<0.001
Nox	28.6 (23.8–33.4)	26.5 (22.8–30.2)	2.1 (−3.9 to 8.1)	0.482	7.0 (−10.2 to 24.2)	0.410
Serum						
Vitamin E	26.7 (22.1–31.3)	29.8 (26.3–33.2)	−3.0 (−8.5 to 2.4)	0.264	−14.4 (−33.1 to 4.3)	0.121
Urate	241 (210–272)	116 (91–141)	125 (86 to 164)	<0.001	173 (37 to 310)	0.016
Proteins	69.0 (66.1−71.9)	72.0 (70.3−73.7)	−3.00 (−6.05 to 0.05)	0.054	−2.25 (−7.48 to 2.98)	0.377
Oxid. proteins	2.85 (2.46–3.23)	4.30 (4.06–4.55)	−1.46 (−1.91 to −1.01)	<0.001	−1.12 (−2.10 to −0.14)	0.027
Conjugated dienes	51.8 (46.7–57.0)	54.8 (49.5–60.1)	−2.9 (−10.2 to 4.3)	0.420	−6.2 (−24.4 to 11.9)	0.486
TRAP	947 (872–1022)	963 (895–1030)	−16 (−115 to 83)	0.751	−89 (−299 to 120)	0.388
TBARS	4.64 (4.33–4.95)	4.60 (4.26–4.93)	0.04 (−0.41 to 0.49)	0.855	−0.78 (−1.95 to 0.38)	0.180
BAL nitrite	0.182 (0.156–0.209)	0.206 (0.183–0.229)	−0.024 (−0.06 to 0.01)	0.176	−0.013 (−0.07 to 0.05)	0.659
			
			Ratio (95% CI)	*P*	Ratio (95% CI)	*P*
			
Plasma glutathione^c^	0.79 (0.51–1.22)	0.89 (0.65–1.23)	0.89 (0.53 to 1.48)	0.636	0.72 (0.15 to 3.54)	0.667
BAL thiols^c^	4.40 (2.69–7.21)	1.48 (1.12–1.95)	2.98 (1.71 to 5.19)	<0.001	2.00 (0.39 to 10.17)	0.378
BAL ascorbate^c^	0.53 (0.29–0.94)	0.79 (0.47–1.34)	0.67 (0.31 to 1.43)	0.293	1.37 (0.16 to 11.98)	0.769
BAL urate^c^	11.18 (7.76–16.11)	6.45 (4.77–8.72)	1.73 (1.10 to 2.73)	0.019	2.43 (0.92 to 6.43)	0.069B
BAL Nox^c^	0.87 (0.77–0.97)	1.34 (1.12–1.60)	0.65 (0.53 to 0.79)	−0.001	1.01 (0.62 to 1.63)	0.979

a Independent samples *t*-test,

b The adjusted comparison (ANCOVA) includes age and FEV_1_ (%) as continuous covariates and gender and smoking (smoker vs non-smoker) as random factors

c Geometric mean (95% CI); the values were logarithmically (ln) transformed before analysis

### Local oxidative stress markers in BAL fluid at baseline

Cancer patients had significantly higher levels of urate (geometric mean 11.18 μmol/L, 95% CI 7.76–16.11 μmol/L vs 6.45 μmol/L, 95% CI 4.77–8.72 μmol/L, *p* = 0.019) and thiols (geometric mean 4.40 μmol/L, 95% CI 2.69–7.21 μmol/L vs 1.48, 95% CI 1.12–1.95 μmol/L, *p*<0.001) and lower levels of Nox (geometric mean 0.87 μmol/L, 95% CI 0.77–0.97 μmol/L vs 1.34 μmol/L, 95% CI 1.12–1.60 μmol/L, *p*<0.001) than controls at baseline. However, after adjustment for age, FEV_1_, gender and smoking, significance was lost for the difference of these markers between the patients and controls (*p* = 0.069, *p* = 0.378 and *p* = 0.979 for BAL urate, thiols or Nox). Nor were there significant differences in the levels of BAL ascorbate or nitrite between LC patients and controls (0.53 μmol/L vs 0.79 μmol/L, *p* = 0.293 and 0.18 μmol/L vs 0.21 μmol/L, *p* = 0.176, respectively). The concentrations of TRAP in BAL fluid samples were undetectable (Table II, Figure 1).

### Systemic oxidative stress markers during and after radiotherapy

**Figure 2:**
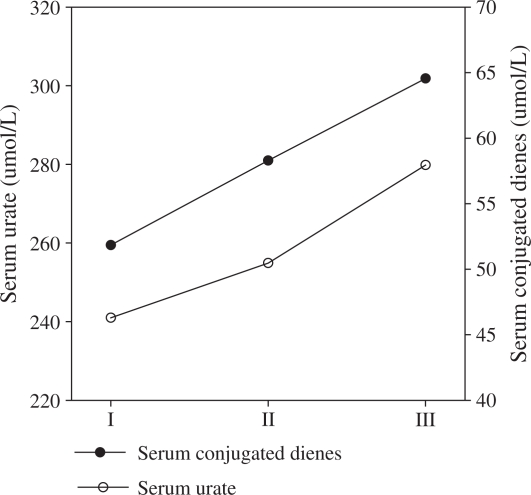
The mean levels of serum conjugated dienes and serum urate (μmol/L) at baseline (I), after 2 weeks of radiotherapy (II) and 3 months after radiotherapy (III).

Although not significant, there was a trend towards an increase in alpha-tocopherol (6.43 vs 8.86 mg/L, *p* = 0.056), urate (220 vs 255 μmol/L, *p* = 0.064) and oxidized proteins (2.64 vs 3.29 μmol/L, *p* = 0.088) after 2 weeks of RT. There was also a slight, but non-significant, reduction in glutathione, thiols, TRAP, TBARS, proteins, Nox and vitamin E. Nonsignificant increases were noted in ascorbate, gamma-tocopherol and conjugated dienes during RT. The levels of nitrite remained unchanged during RT (Figure 2).

Three months after RT, the levels of urate (214 vs 280 μmol/L, *p* = 0.044), conjugated dienes (53.4 vs 64.6 μmol/L, *p* = 0.034) and TBARS (4.58 vs 5.64 μmol/L, *p* = 0.004) had risen significantly. A nearly significant increase was noted in alpha-tocopherol (*p* = 0.055). The changes in the levels of other antioxidants and markers of oxidative and nitrosative stress 3 months after RT were non-significant.

### Local oxidative stress markers in BAL fluid during radiotherapy

There was an almost significant increase in urate (geometric mean 7.30 μmol/L vs 13.91 μmol/L, *p* = 0.083) and thiols (geometric mean 3.34 μmol/L vs 4.85 μmol/L, *p* = 0.069) after 20 Gy of RT compared to baseline. No notable changes took place for BAL ascorbate, nitrite or Nox during RT.

### Oxidative stress markers and lung cancer stage

Higher BAL thiol (6.29 μmol/L vs 2.54 μmol/L, *p* = 0.063) and BAL nitrite (0.21 μmol/L vs 0.15 μmol/L, *p* = 0.021) levels and lower serum oxidized proteins (2.36 μmol/L vs 3.39 μmol/L, *p* = 0.005) and serum TBARS (4.37 μmol/L vs 4.94 μmol/L, *p* = 0.067) levels were recorded in stages I–III compared to stage IV disease.

### Association between oxidative stress markers, adverse events and response to RT

Overall toxicity during RT was mild and none of the patients experienced any serious adverse events. Thirteen (65%) patients experienced gr I/II adverse events during RT: esophagitis (*n* = 9), cough (*n* = 4), fatigue (*n* = 4) and fever (*n* = 4). Five patients (25%) developed symptomatic radiation pneumonitis. The occurrence of adverse events during RT was not significantly associated with any baseline oxidative stress marker levels.

Twenty-five per cent of the LC patients achieved complete and 56.3% partial response to RT, whereas 6.3% had no change and 12.5% had progressive disease. The response to treatment was not significantly associated with any baseline oxidative stress marker levels.

### Association between oxidative stress markers and overall survival

**Figure 3:**
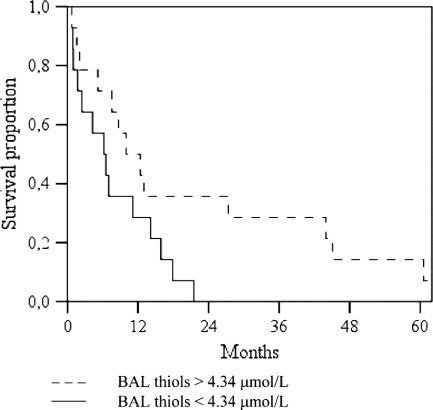
Kaplan-Meier survival curves for patients with baseline BAL thiols >4.34 μmol/L (*n* = 14) vs patients with BAL thiols <4.34 μmol/L *n* = 14). Log-rank test *p* = 0.051.

The planned follow-up time of the patient group was 72 months. None of the patients was lost to follow-up. At the end of the study, one patient (3%) was alive and 35 (97%) had died. The median survival time of the patients was 9.9 months (95% CI 5.4–14.4 months). The survival time tended to be longer when the patient's baseline BAL thiol levels were above the median concentration of 4.34 μmol/L (9.9 months vs 6.1. months, *p* = 0.051) (Figure 3).

### Associations between oxidative stress markers and other demographics

There were no significant differences in oxidative stress markers between smokers and non-smokers among the patients. However, among the controls, smokers had significantly higher levels of BAL thiol (2.73 vs 1.47 μmol/L, *p* = 0.022), serum urate (177 vs 106 μmol/L, *p* = 0.035), serum TBARS (5.06 vs 4.36 μmol/L, *p* = 0.042) and serum nitrite (0.25 vs 0.14 μmol/L, *p* = 0.017) and lower levels of plasma ascorbate (59 vs 88 μmol/L, *p* = 0.011). Among the controls, the number of pack years smoked correlated negatively with plasma ascorbic acid (*R* = −0.452, *p* = 0.014).

There was a trend favouring an association between weight loss prior to diagnosis and overall survival, but this finding lacked statistical significance (*p* = 0.769). If patients had lost weight less than 2 kg prior to lung cancer diagnosis, the median overall survival was 12.4 months (95% CI 9.2–15.6 months); if weight loss was 3–5 kg median survival was 8.7 months (95% CI 4.0–13.4 months) and if weight loss was more than 5 kg, median survival was 3.8 months (95% CI 0–11.5 months).

**Figure 4:**
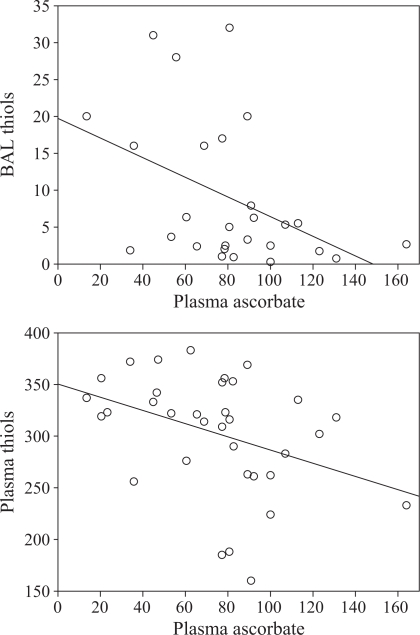
Scatterplots and regression lines for associations between patients plasma ascorbate (μmol/L) vs BAL thiols (μmol/L), Spearman *R* = −0.377, *p* = 0.052 and plasma thiols (μmol/L), *R* = −0.446, *p* = 0.008. One outlier with plasma ascorbate 60.5 vs BAL thiols 11.9 is outside the figure.

There were significant positive correlations between BAL ascorbic acid and plasma ascorbic acid (*R* = 0.342, *p* = 0.005), BAL thiols and both serum urate (*R* = 0.481, *p* = 0.001) and serum alpha-tocopherol (*R* = 0.714, *p* = 0.031) and a negative correlation between plasma ascorbic acid and both plasma and BAL thiols at baseline (*R* = −0.446, *p* = 0.008 and *R* = −0.377, *p* = 0.052, respectively) (Figure 4).

## Discussion

This study shows that lung cancer is associated with enhanced circulating concentrations of urate and nitrite (*p* < 0.001 for both), which is in accordance with previous findings [37,38]. Urate, which is the end product of purine metabolism, is one of the major antioxidants in human plasma and it may play an essential role in protecting cells against free-radical induced damage. Thus, elevated levels of urate may signify a compensatory mechanism to oxidative stress [39]. It is also shown that both cancer and RT are associated with increased oxidative damage to DNA [40,41] and thus hyperuricemia might be also partly due to increased purine metabolism through the effects of xanthine oxidase as a consequence of RNA-DNA breakdown [42].

Nitric oxide (·NO) is involved in many physiological processes and has an extremely short half-life [43]. However, the blood ·NO level does not necessarily reflect the ·NO status of the tissues. In plasma and other physiological fluids ·NO is oxidized to nitrite, whereas in the whole blood ·NO and nitrite are oxidized to nitrate [44]. Increased production of nitric oxide may protect the cells from oxidative stress and this might explain the elevated levels of nitrite among LC patients compared to controls (*p* < 0.001, Table II) [45]. On the other hand, production of a potent oxidant and cytotoxic molecule, peroxynitrite in the reaction of ·NO with the superoxide anion may lead to increased biochemical reactivity and a wide range of damaging effects.

Increased free radical production, decreased activity of the antioxidant defense mechanisms or enhanced consumption of antioxidants lead to oxidative stress [46]. The total antioxidant capacity has been used to measure oxidative stress in whole body [45,47]. In a previous study we reported significantly lower total peroxyl radical trapping antioxidant potential (TRAP) levels in LC patients compared to healthy controls [48]. Although also the present study noted decreased levels of TRAP in LC patients compared to controls (Table II), the difference between the two groups was not significant. This is in agreement with a previous study [49]. Yet, the known components of TRAP, besides urate, tended to be lower in the LC group than controls: protein SH-groups (thiols) (*p* < 0.001, *p* = 0.651 after adjustment for age, FEV_1_, gender and smoking), ascorbic acid (*p* = 0.53, *p* = 0.753 after adjustment) and vitamin E (*p* = 0.26, *p* = 0.121 after adjustment). We also recorded a significant positive correlation between plasma TRAP and serum urate, which corroborates a previous similar observation [50].

The antioxidant levels in BAL fluid of lung cancer patients have not been the subject of very much research. After adjustment for age, FEV_1_, gender and smoking, no significant differences were noted between LC patients and non-cancer controls in BAL fluid oxidative stress markers, although LC patients tended to have higher levels of BAL urate (*p* = 0.069). As bronchoscopy was performed on the control patients because of prolonged cough, it is possible that they had altered mucus secretion due to hypertrophy in mucus secreting glandula caused by chronic, hyperplastic bronchitis appearing as prolonged cough. This may partly explain why no differences were seen in BAL oxidative stress markers between LC patients and non-cancer controls. The levels of BAL ascorbate seen in this study are in conformity with previous studies [7,12,51]. The levels of BAL urate are higher than reported in some previous studies [7,12,51]; however, in alignment with the levels obtained of lung transplant recipients [52]. The method we used to analyse BAL and plasma thiols measures all small molecular weight as well as protein thiols, which explains the higher levels compared to studies reporting only glutathione/reduced glutathione levels [12,13].

We observed a tendency of BAL urate (*p* = 0.083) and thiols (*p* = 0.069) to rise during RT, a finding not reported previously. Although not significant, the levels of plasma alpha-tocopherol (*p* = 0.056), serum urate (*p* = 0.064) and serum oxidized proteins (*p* = 0.088) tended to increase after 2 weeks of RT. The elevated levels of urate both locally and systemically during RT might be attributed to enhanced cell necrosis following RT; it is known that urate is released from dying cells [46]. This increase in urate during RT is in accordance with previous findings [48]. It has been shown that RT also produces thiol radicals, which can react with ascorbic acid or ·NO [46]. The elevated levels of BAL urate might also be attributed to the movement of urate onto the lung surface to protect against oxidative stress caused by RT [12]. The elevation of oxidized proteins levels during RT might be a causal factor in an early event of oxidative endothelial cell damage and also a marker of enhanced RT-related oxidative stress [53]. Aside from urate, the changes in BAL fluid antioxidant levels during RT seem to be independent of systemic changes in these markers.

Lipid peroxidation might be one of the main causes of damage during RT and previous studies have reported increased lipid peroxidation marker levels during RT [54,55]. RT is also known to cause oxidation of membrane protein SH-groups, which may explain the present finding of elevated levels of serum oxidized proteins and BAL thiols during RT [56].

Thiobarbituric-acid-reactive substances (TBARS) are considered to be markers of lipid peroxidation in tissues and the plasma, although rather unspecific [46,57–59]. During the early phase of lipid peroxidation the double bonds of polyunsaturated fatty acids (PUFAs) are rearranged and conjugated dienes are formed. Conjugated dienes have been widely studied as an index of lipid peroxidation and are less sensitive to the compensatory antioxidant mechanisms than the other lipid peroxidation markers [59,60]. The significantly elevated levels of serum conjugated dienes (*p* = 0.034) and TBARS (*p* = 0.004) 3 months after RT imply that the main antioxidants scavenger systems are consumed during RT, leading to enhanced lipid peroxidation after RT [45]. Previous studies have shown that the onset of lipid peroxidation caused by RT may be delayed [61]. In the present study lower baseline serum TBARS levels were measured in limited disease (stages I/II/III) compared to extensive disease (stage IV, *p* = 0.067), which is supported by a previous study reporting reduced plasma malondialdehyde concentration with decreasing tumour size [62].

To our knowledge, this is the largest study so far to evaluate different antioxidants and parameters of oxidative and nitrosative stress in lung cancer patients and during RT for lung cancer. No previous clinical follow-up studies have been performed that have recorded oxidative stress markers in relation to response to RT and overall survival of LC patients. We found no associations between any of the oxidative stress marker baseline levels and adverse events during or after RT or response to treatment. However, a borderline significant association was recorded between higher BAL thiol levels and longer overall survival. A recent study observed that head and neck carcinoma patients who have a higher than median concentration of glutathione in plasma survive longer [63]. Measuring also the ratio of oxidized glutathione to reduced glutathione in this study would have added beneficial information to the hypothesis that increased glutathione is associated with overall survival; this is one limitation of our finding. We also noted that the LC stage was associated with baseline BAL thiol levels: higher local thiol levels were seen in stage I–III LC compared to stage IV LC (*p* = 0.063). Although these associations are of borderline statistical significance, it is also possible that the longer survival of some of the patients in this study is related to limited stage of the disease. Obviously, larger studies are needed to discriminate cause and effect in this respect.

## Conclusions

This study supports strongly the hypothesis that LC is associated with increased oxidative stress. The findings suggest that antioxidant responses may serve as a protective mechanism against production of ROS during RT The cellular damage caused by RT may also result in release of intracellular antioxidative substances. This study indicates that oxidative stress caused by RT may be counterbalanced by local lung antioxidant systems; however, notable lipid peroxida-tion does occur after RT. None of the examined oxidative stress markers prognosticated adverse events during RT or the response to treatment. However, the results imply that BAL thiols may be associated with overall survival of LC patients.

## Abbreviations

BAL: bronchoalveolar lavage
LC: lung cancer
RT: radiotherapy
